# 

**Published:** 1877-12-20

**Authors:** 


ORIGINAL AND SELECTED ARTICLES—

The Duty of tne Doctor to the Dying, ........ 311
Continued Menstruation During Pregnancy ........ 313
Fumigation in Syphilis ........ 314
Plaster-of-Paris Bandage, ........ 317
Notes on Hospital Practice ........ 320

ABSTRACTS AND GLEANINGS—

Case of Empyema following Pneumonia—Early Aspiration—Recovery, 322; Teeth at Birth—Extraction—Hemorrhage—Death, 323; Successfuc Removal of a Large Fibroid Uterus with Both Ovaries, 324; Fecundation of the Hen's Egg in the Oviducts, 324; Rheumatic Dismenorrhea, 325; Strabismus, 326; Signs by which Phthisis is Recognized in its Earliest Stages without the aid of Physical Examination of the Chest, 326; Influence of Drinking-Water on the Biliary Secretion, 327; A Female Chemist, 327; Lemon - Juice in Rheumatism, 327; Japanese Therapeutics, 328; Gelseminum and Quinine, 328; Whitlow of the Thumb, 329; Arsenical Antidotes, 329; The Effects of Iron upon the Blood, 329; Syphilitic Fathers and Healthy Children, 329; Chenopodium Anthelminticum for Anasarca, 329; Treatment of Boils, Carbuncles, Etc., 330; Morphia in Catheterism, 330; Brewery Grains as Food for Cows, 330; Arsenic in Albuminuria, 330; Hydrophobia During Pregnancy, 330; Fracture of the Femur—Buck's Method, 331; For Salivation, 331; Iodoform in Typhoid Fever, 332; Quinia in Small Doses in Malarial Diseases, 332; Solution of Quinine for Hypodermic Use, 332; Prevention of Lead Poisoning, 332; Kerosene Liniment, 332; Vaccine Virus, 332; Sclerotic Acid, 332.

PRACTICAL NOTES AND FORMULÆ—

How to Take Castor Oil Without Tasting It 333; Chloral and Alcohol, 333; Carbollic Acid in Diarrhœa, 333; Freckles, 333; Lime in the Eye, 333; Burns, 333; Elixir of Wild Cherry Bark, 334; Ricord's Cough-pills, 334; Rum Stomach or Flatulent Dyspepsia, 334; Canada Balsam as an Excipient for Pills, 334; Shaving Soap, 334; Elixir Chloroformi, 334; Crysophanic Acid in Psoriasis, 334; Hair Wash, 334; Ranula, 334; Copabia Resin in Ascites, 334; Enlarged Tonsils, 335; Nitric Acid, Senna and Taraxicum as a Liver Regulator, 335; Syrup of Hypophosphate of Sodium, 335; Chloric Ether, 335; For Bed Sores, 335; For Vermin, 335; Chronic Hepatic Affections, 335; Salicylic Acid in Pertussis, 335; Tonic, Laxative and Anodyne, 335; Spiritus Chloroformi, 335; Puerperal Fever, 335; Carbolic Acid Spray, 335; Red Lotion of Hospitals, 336; Tannin Gargle, 336; Gargle for Foul Ulcers, 336; Iodine Inhalation, 336; To Check Secretion of Milk, 336; To Keep Leeches, 336; Epileptic Hysteria, 336; Turpentine Inhalation, 336; For Tape Worm, 336; For Chronic Rheumatism, 336.

SCIENTIFIC ITEMS—

Experiments on the Circulation in the Brain, 336; Telephony, 336; Electric Plant, 336.

EDITORIAL AND MISCELLANEOUS—

Notice—Last Call, 337; Our Next Volume, 337; Correction, 337; Please Read, 337; The Receding Year, 337; Strange but True, 338; Sulphate of Cinchonidia, 338; Advertisements for 1878, 338; Purchasing Agency, 339.

BOOK NOTICES.

Specific Meditation and Specific Medicines, 339; An index of Disease and Their Treatment. 340.

